# 1083. Phase 1b Results of Pharmacokinetics, Pharmacodynamics, and Safety for LBP-EC01, a CRISPR-Cas3 Enhanced Bacteriophage Cocktail Targeting *Escherichia coli* that Cause Urinary Tract Infections

**DOI:** 10.1093/ofid/ofab466.1277

**Published:** 2021-12-04

**Authors:** Paul Kim, Ana Sanchez, Jamie Kime, Dave ousterout

**Affiliations:** Locus Biosciences, Morrisville, North Carolina

## Abstract

**Background:**

LBP-EC01 is the first CRISPR-engineered bacteriophage product to successfully complete Phase 1b testing in a clinical program designed to address infections caused by E. coli initially targeting urinary tract infections (UTIs). Thirty-six subjects were enrolled in this randomized, double blind study to assess the safety, tolerability, pharmacokinetics and pharmacodynamics of LBP-EC01 in patients with lower urinary tract colonization caused by E. coli.

Methods

**Results:**

No drug-related Treatment Emergent Adverse Events (TEAEs) were observed. All nondrug related TEAEs were Grade 2 or below and there were no tolerability signals associated with LBP-EC01.A modified ITT (mITT) population was defined to assess PK in subjects treated with LBP-EC01 (n=17). Subjects were removed from the PK analysis who had missing or insufficient colonization at baseline (n=3), were exposed to antibiotics during screening or study conduct (n=3) or exhibited a non-drug related SAE (n=1). Of these subjects, 12 were found to be sensitive to LBP-EC01 and of these, 10 (83%) showed phage amplification. A PD analysis compared the mITT populations of LBP-EC01 vs. placebo (n=6) and showed that the LBP-EC01 arm had a decrease in E. coli that was greatest within 24 hours of initial treatment and remained below baseline across the entire treatment period. The placebo arm showed increased levels of E. coli and higher variability over the treatment period. An average difference of 2-3 log (100x to 1,000x) existed in urine E. coli concentration (CFU/mL) between the LBP-EC01 and placebo arms across the duration of the treatment period.

**Conclusion:**

LBP-EC01 has proved to be safe and well-tolerated in this Phase 1b study of subjects colonized by E. coli. In addition, phage amplification was observed in patients with E. coli isolates sensitive to LBP-EC01, demonstrating a clear proof of mechanism. Finally, the apparent difference in PD effect between LBP-EC01 and placebo which was irrespective of MDR status, provides promise that LBP-EC01 may someday be an excellent option for patients suffering from UTIs caused by E. coli, especially in strains that are resistant to conventional antibiotics.

**Disclosures:**

**Dave ousterout, PhD**, **InceptorBio** (Advisor or Review Panel member, Shareholder)**Locus Biosciences** (Employee, Shareholder)

